# Investigation of Prognostic Factors for Intense Pulsed Light Treatment with a Vascular Filter in Patients with Moderate or Severe Meibomian Gland Dysfunction

**DOI:** 10.3390/jcm11164724

**Published:** 2022-08-12

**Authors:** Yunhan Lee, Joon Hyuck Jang, Sanghyu Nam, Koeun Lee, Jin Kim, Jae Yong Kim, Hungwon Tchah, Hun Lee

**Affiliations:** Department of Ophthalmology, Asan Medical Center, University of Ulsan College of Medicine, Seoul 05505, Korea

**Keywords:** intense pulsed light, vascular filter, MMP-9, meibomian gland dysfunction, telangiectasis, age, meibum consistency

## Abstract

We aimed to investigate the prognostic factors for, and treatment efficacy of, intense pulsed light (IPL) treatment with a vascular filter in patients with moderate or severe meibomian gland dysfunction (MGD). In this retrospective observational study, 58 moderate or severe MGD patients who underwent IPL treatment with a vascular filter were enrolled. IPL treatment was administered to the upper and lower eyelids four times at two-week intervals. At baseline, and four weeks after IPL, we evaluated the matrix metalloproteinase (MMP)-9 expression levels, tear break-up times (TBUT), ocular surface staining scores, lid margin telangiectasias, and meibomian gland characteristics. The subjective symptoms and adverse effects were reviewed and recorded. Regression analyses were performed to explore the prognostic factors affecting clinical outcomes. IPL treatment using a vascular filter led to improvements in the TBUT, ocular surface staining score, meibomian gland grade, meibum quality and consistency, lid margin telangiectasia, and symptom score (all *p* < 0.001). Furthermore, the positivity rate (90.2% to 70.6%, *p* = 0.013) and expression levels (1.92 ± 1.18 to 1.24 ± 1.18, *p* < 0.001) of tear MMP-9 improved after the IPL treatment. In multivariate logistic regression analysis, a young age (odds ratio = 0.867, *p* = 0.007) and a toothpaste-like consistency in the upper lid (odds ratio = 8.449, *p* = 0.046) were associated with improvements in the meibomian gland grade. No adverse effects were detected. IPL with a vascular filter is a safe and effective treatment for moderate and severe MGD. Age and the meibum consistency in the upper lid are important prognostic factors.

## 1. Introduction

Dry eye syndrome arises from multiple etiologies including meibomian gland dysfunction (MGD), which is a dysfunction due to an obstruction of the gland from the distal end [1,2]. MGD is accompanied by chronic inflammation and diffuse structural abnormalities of the meibomian gland (MG) [1]. Furthermore, inflammatory mediators, which are key factors in dry eye syndrome, are secreted from abnormal vessels on the lid margin, i.e., telangiectasia, around the MG [1,3].

The traditional treatment options for MGD can be separated into lubrication, inflammation control using topical eyedrops and oral antibiotics, mechanical alleviation of terminal duct obstruction, and omega-3 fatty acid supplementation [4,5,6,7,8,9,10]. However, these treatments provide only short-term relief for the symptomology [11,12]. Therefore, a need for alternative treatments has emerged, especially treatments with good long-term effects on clinical MGD outcomes. Intense pulsed light (IPL) treatment is a well-known and effective treatment modality, even for refractory-type MGD [13,14,15,16,17]. IPL is a noncoherent polychromatic light source with a wavelength range of 400–1200 nm [18]. When this light is absorbed by chromophores, such as melanin, hemoglobin, and water, it can induce selective photothermolysis [16]. IPL affects MGD through photothrombosis of the lid telangiectasia, photomodulation of cytokines such as the matrix metalloproteinase (MMP) and reactive oxygen species levels, liquefaction of the meibum, and Demodex follicles mite eradication [19]. The therapeutic ranges of the wavelength spectrum can be selected by using specific filters [18]. IPL treatment using an acne filter was subsequently applied because supplementary treatment was essential to improve the telangiectasia. The acne filter, as a notch filter, is able to selectively emit light of 400–600 nm and 800–1200 nm by blocking the 600–800 nm portion of the spectrum. The 600–800 nm wavelength light is mainly absorbed by melanin, inducing pain. On the contrary, the hemoglobin in the superficial and deep tissue vasculatures absorbs light of 400–600 nm and 800–1200 nm, respectively [20]. The energy from the IPL device can be focused on a vascular structure, especially the lid margin telangiectasia, and destroy the abnormal vascular lesion around the lid margin. There was a significant improvement in the lid margin telangiectasia of both the upper and lower eyelids four weeks after IPL treatment using an acne filter; subjective symptoms and clinical signs improved, as well [20]. Similar to the acne filter, the vascular filter is a notched filter that can emit light of shorter wavelengths (530–650 nm) to target the porphyrins and hemoglobin in superficial blood vessels, and light of longer wavelengths (900–1200 nm) to target the hemoglobin in deep blood vessels. IPL treatment using a vascular filter can allow the energy to be focused on the vascular structure. In vascular filters, rejected bands of 650–900 nm filter out the wavelengths for which the extinction coefficient is not high enough for the oxyhemoglobin (HbO_2_) or deoxyhemoglobin (Hb). This preserves the light intensity only in spectral windows for which there is sufficient HbO_2_ absorption to support suitable photothermal conversion to methemoglobin (MetHb). IPL treatment with a vascular filter has special effects, not only because of the specific filter but also because of a system called advanced optimal pulse technology (AOPT; personalized multi-sequential pulsing). This technology separates every particle of light emitted from the IPL into three pulses, and each pulse can be modulated into a specific strength and duration. When vascular filter-assisted IPL meets the AOPT, there are three resulting advantages. First, HbO_2_ is converted to Hb, which is then converted to MetHb during the first sub-pulse. Second, denaturation of the targeted tissue occurs during the intervals between each pulse, allowing it to absorb 3–4 times more energy. Third, because of this denaturation, the maximal thermal effect can be obtained with less pulse power and minimal tissue damage. Pulsed light over 900 nm reaches the deeper vasculature for complete thrombosis to deliver thermal energy to MetHb, with a maximal extinction coefficient in 900–1200 nm wavelengths.

In the present study, we aimed to evaluate the therapeutic application of IPL treatment using a vascular filter in patients with moderate or severe MGD.

## 2. Materials and Methods

This study was conducted with the approval of the Institutional Review Board of the Asan Medical Center and the University of Ulsan College of Medicine, Seoul, South Korea (2021-0582), which waived the need for informed consent. The study adhered to the tenets of the Declaration of Helsinki and followed good clinical practice guidelines. Patient consent was waived due to the retrospective nature of this study.

Patients over 19 years old with moderate or severe MGD were retrospectively enrolled at the IPL Dry Eye Clinic after a washout period of four weeks. The severity of the MGD was staged according to criteria stated in the MGD International Workshop by the Tear Film and Ocular Surface Society [21]. Patients who had undergone previous ocular surgery, had eyelid structural anomalies, an ocular infection history, inflammation unrelated to dry eye, autoimmune disease, glaucoma with topical medication, ocular allergy, a history of using contact lenses during the study period, semi-permanent makeup or tattoos, hyperpigmentation of eyelids, a history of skin treatment within 2 months prior to the study, or were pregnant or lactating, were excluded.

A total of 58 eyes in 58 patients underwent IPL treatment using a vascular filter (four sessions with an interval of 2–3 weeks between them) by a single trained physician (HL). Prior to starting the treatment sessions, every patient underwent Fitzpatrick skin typing, and adjustment of the IPL system (M22; Lumenis, Yokneam, Israel) was performed for a specific setting (fluence delivering in a 6-5-4 J/cm^2^ decrement pattern) [22]. For lower eyelid treatment, topical anesthesia was applied first, followed by an ultrasonic gel and metal spatula coverage, while both eyelids were closed. After exposure of the lower eyelid margin, the IPL probe was applied in a slightly downward position with tractional force on the lower eyelid to increase the contact surface between the probe and the lid margin [20]. Patients received up to 13 light pulses with a slightly overlapping area of application, from the left preauricular area, across the cheeks and nose, to the right preauricular area [17,23]. For the upper lid IPL treatment, a metal spatula was carefully placed into the upper conjunctival fornix and slightly tented to minimize ocular damage. Patients received 2–3 light pulses on each side of the upper eyelid [20].

Examinations were performed before the first treatment and four weeks after the last treatment. Recorded clinical signs included the extracellular MMP-9 levels (using the InflammaDry immunoassay device; Rapid Pathogen Screening, Inc., Sarasota, FL, USA), tear film break-up time (TBUT), ocular staining scores of the cornea and conjunctiva [Sjögren’s International Clinical Collaborative Alliance (SICCA) and Oxford score], Schirmer’s test I without topical anesthesia, lid margin abnormalities, and meibum characteristics [23,24,25].

In the InflammaDry immunoassay, one blue line in the control zone with one red line in the result zone indicates a positive test result, whereas one blue line without a red line indicates a negative test result. A red line in the result zone indicates concentrations of MMP-9 over 40 ng/mL (strong positive, positive, or weak positive). Across the grading scale, we applied semi-quantitative interpretations of the ocular surface inflammation severity. Because of the proportional increases in MMP-9 concentrations with signal strength, the more vivid the color in the red zone, the higher the MMP-9 levels (0 = none; 1 = trace; 2 = weak positive; 3 = positive; and 4 = strong positive) [17,20]. The eye with a more severe MG grade was used for the MMP-9 measurement. When both eyes showed the same grade of MG, the right eye was chosen.

The lid margin telangiectasia (0 = no or slight redness in the lid margin conjunctiva and no telangiectasia crossing the MG orifices; 1 = redness in the lid margin conjunctiva and no telangiectasia crossing the MG orifices; 2 = redness in the lid margin conjunctiva and telangiectasia crossing the MG orifices with a distribution of less than half the full length of the lid; and 3 = redness in the lid margin conjunctiva and telangiectasia crossing the MG orifices with a distribution of half or more of the full length of the lid) was assessed and scored for both the upper and lower lids [26]. The MG grade was measured semi-quantitatively after applying digital pressure on the upper tarsus (grade 0 = clear meibum easily expressed; grade 1 = cloudy meibum expressed with mild pressure; grade 2 = cloudy meibum expressed with more than moderate pressure; and grade 3 = no meibum expression with firm pressure) [27]. An evaluation of the meibum color (1 = clear; 2 = cloudy; and 3 = yellow) and consistency (1 = oily; 2 = creamy; and 3 = toothpaste-like) was performed with the MG with expressor forceps on the upper and lower lids [23]. The ocular irritation symptom score (ocular discomfort, itching, and photophobia with limitations of activities) on a scale of 0 (no symptom) to 3 (severe) was used to assess subjective symptomology.

Sequential examination of the clinical signs was carried out following the same order in every patient: first with the MMP-9 InflammaDry immunoassay, then Schirmer’s test I, TBUT, the ocular staining scores in the cornea and conjunctiva with fluorescein, and finally, an examination of the lid margins and MG. After completing sequential physical examinations, patients responded to a questionnaire about their ocular irritation symptoms [17,20]. At each visit, the best spectacle-corrected visual acuity (BCVA) and intraocular pressure (IOP) were measured. Possible adverse events were examined via a slit-lamp examination at every visit. Any dermatologic adverse events were also evaluated and recorded, such as redness, depigmentation, blistering, or swelling.

### Statistical Analysis

Statistical analyses were performed using SPSS software version 25.0 (IBM Corp., Armonk, NY, USA). The normality of the data was analyzed using the Shapiro–Wilk test. Paired *t*-testing was performed to compare the clinical parameters before, and four weeks after, the IPL treatment. The McNemar’s test and Fisher’s exact test were used to compare the results of the MMP-9 immunoassay before, and four weeks after, treatment. Based on differences in the MG grades before and after the IPL treatments, patients were classified into two subgroups: (the non-responder group—no difference in the MG grade, and the responder group—an improvement in the MG grade). Independent *t*-tests were performed to compare the clinical parameters between the non-responder and responder groups. Univariate and multivariate logistic regression analyses were used to identify factors affecting improvements in the MG grade. A *p* value of less than 0.05 was considered statistically significant.

## 3. Results

A total of 58 eyes in 58 patients (42 female) with moderate or severe MGD were included in this study. The mean age was 63.2 ± 9.4 years (range 37–80). The clinical signs are presented in Table 1.

A significant increase in the TBUT (1.58 ± 0.86 to 3.88 ± 1.35, *p* < 0.001) and significant decreases in the SICCA score (5.98 ± 2.34 to 1.98 ± 1.56, *p* < 0.001) and the Oxford score (2.33 ± 1.15 to 1.03 ± 0.18, *p* < 0.001) were observed four weeks after IPL treatment. There was a significant improvement in the ocular irritation symptom score (2.98 ± 0.13 to 1.17 ± 0.38, *p* < 0.001).

Table 2 shows the lid margin telangiectasia and meibum findings on slit-lamp examination and the ocular surface MMP-9 expression levels before and four weeks after the last IPL treatment.

A significant improvement in the lid margin telangiectasia was detected at four weeks after the last IPL treatment in both the upper and lower eyelids (upper: 2.71 ± 0.46 to 1.36 ± 0.52, *p* < 0.001; lower: 2.81 ± 0.40 to 1.45 ± 0.54, *p* < 0.001). Along with an improvement in the MG characteristics, such as the meibum color, meibum consistency, and MG grade, the positivity rate (90.2% to 70.6%, *p* = 0.013) and expression levels of MMP-9 (1.92 ± 1.18 to 1.24 ± 1.18, *p* < 0.001) on the ocular surface showed significant improvements.

According to the subgroup analysis, there were significant differences in several baseline parameters between the non-responder and responder groups (Table 3).

The meibum consistency in the upper and lower lids (2.00 ± 0.53, 2.25 ± 0.71, respectively, in the non-responder group versus 2.68 ± 0.47, 2.86 ± 0.35, respectively, in the responder group; *p* = 0.008) and the SICCA staining scores (4.88 ± 2.42 in the non-responder group versus 6.16 ± 2.30 in the responder group, *p* = 0.015) were worse in the responder group. The MMP-9 level was higher (1.00 ± 1.07 in the non-responder group versus 2.09 ± 1.13 in the responder group, *p* = 0.015), and the mean age was younger (68.5 ± 9.4 years in the non-responder group versus 62.4 ± 9.3 years in the responder group, *p* = 0.008).

Table 4 and Table 5 show the results of our univariate and multivariate analyses with logistic regression modeling. In the univariate analysis, a younger age (OR = 0.919, *p* = 0.008), more severe baseline SICCA staining score (OR = 1.298, *p* = 0.015), more toothpaste-like secretion of meibum from upper and lower lids (OR = 15.782 and 9.611, *p* = 0.010 and 0.004, respectively), and higher baseline MMP-9 level (OR = 2.856, *p* = 0.027) were significantly associated with effective IPL treatment using a vascular filter. Moreover, in the multivariate analysis, age (OR = 0.905, *p* = 0.010) and meibum consistency in the upper lid (OR = 10.826, *p* = 0.010) were significant factors affecting improvements in the MG grade. There was no significant change in BCVA or IOP after four sessions of IPL treatment. IPL treatment showed no adverse effects on intraocular structures, including the iris or eyelid skin.

## 4. Discussion

IPL treatment with a vascular filter is an effective and safe treatment modality for patients with moderate to severe MGD. It improves clinical signs such as lid margin telangiectasia, MG functionality, and tear MMP-9 expression levels, as well as relieving subjective symptoms. No significant ocular or dermatologic adverse events were recorded. IPL treatment has emerged as an alternative treatment for MGD because of its long-term clinical efficacy when compared with conventional treatments [11,12,14,17,20,28]. The IPL device itself generates a wide spectrum of light energy, and most chromophores, including hemoglobin, melanin, and water, absorb broad spectrum wavelengths. Each chromophore has different absorption rates according to wavelength. For example, hemoglobin has the highest absorption coefficient in light, with a wavelength of 400–600 nm, maintains the second peak absorption coefficient in wavelengths over 800 nm, and has the lowest absorption in wavelengths of 600–800 nm [29,30].

Using specific filters, physicians can select and adjust the range of wavelengths within each pulse according to the clinical setting. In a previous study, lower eyelid IPL treatment with a 590 nm filter was applied for moderate or severe MGD patients. It was effective in significantly improving several MGD objective signs and subjective symptoms; however, the eyelid margin telangiectasia did not show improvements in either eyelid region [17]. A subsequent study using an acne filter, a notch filter, demonstrated the efficacy and safety of an acne filter for the treatment of moderate and severe MGD. This was especially true for the lid margin telangiectasia and ocular surface MMP-9 expression levels [20].

We investigated the effect of a different notch filter—a vascular filter—on the clinical signs and subjective symptoms in patients with moderate or severe MGD. After IPL treatment using a vascular filter, the TBUT, SICCA, and Oxford scores improved significantly, as did the subjective symptoms. The color, consistency, and MG grade also improved. Most importantly, there was a significant improvement in the eyelid margin telangiectasia, which can prevent inflammatory mediator secretion and bacterial overgrowth [15]. MMP-9 mediates ocular surface inflammation by augmenting the inflammatory response [31,32]. Significant improvements in the positivity rates and MMP-9 expression levels were observed, which could explain improvements in the clinical signs and symptoms. In one study that evaluated the effect of an intra-MG bevacizumab injection on the lid margin telangiectasia in MGD patients, the lid margin telangiectasia was improved significantly, subsequently resulting in improvements in TBUT, corneal staining scores, and symptoms [33]. In accordance with previous studies on IPL, Schirmer’s test was not improved in the present study [23,30,34]. Regardless of the filter types, IPL treatment itself cannot affect lacrimal gland secretions; rather, it influences the MG alone.

In one recent study investigating the prognostic factors of IPL treatment using a 590 nm filter, the IPL responder group showed a younger age, longer TBUT, less meibomian gland loss, and lower meiboscores before the IPL treatment [35]. In our study, the responder group showing an improvement in the MG grade had a younger mean age, but the TBUT was not different between the non-responder and responder groups. Interestingly, the responder group had a worse SICCA staining score, toothpaste-like thick meibum consistency, and higher MMP-9 expression levels. In the univariate analysis, these variables were associated with effective IPL treatment using a vascular filter, as evidenced by improvements in the MG grade. Our results suggest that a more severe baseline MGD was noted in the responder group, unlike in the previous study showing a lower baseline meiboscore in the responder group. These conflicting results can be mainly attributed to the filter type. Unlike a 590 nm filter, IPL treatment with a vascular filter can efficiently deliver energy into superficial and deep vessels and, via AOPT, more targeted thrombosis of abnormal vessels is possible. These features contribute to our assumption that IPL treatment with a vascular filter has more prominent effects in patients with moderate or severe MGD. Another study demonstrated that the baseline MG grade was an important factor associated with effective IPL treatment [36]. It revealed that moderate MG grade had the highest OR for effective IPL treatment, followed by mild and severe MG grades. These results are based on the different IPL instrument (E-SWIN, Paris, France), making a direct comparison to our study inappropriate. With regard to the MMP-9 levels, we assumed that higher MMP-9 levels indicate more abnormal superficial and deep vasculature of the eyelid. More targeted thrombosis of the superficial and deep vasculature via IPL treatment with a vascular filter contributed to higher MMP-9 expression levels in the responder group versus the non-responder group.

Vascular filters can allow for the minimization of the amount of light absorbed by melanin pigments by preventing the emission of light of 650–900 nm in wavelength. Theoretically, vascular filters can enhance the therapeutic effect on the lid margin telangiectasia by maximizing the amount of light absorbed by the hemoglobin. The light energy absorbed by the hemoglobin is converted to heat energy, raising the temperature of the vessel center to 80–90 °C, subsequently causing thrombosis of the superficial blood vessels [16]. As a result, a key reservoir of inflammatory mediators is destroyed, thus eliminating a main source of inflammation from the MG and the eyelids [16]. With regard to the acne filter, light of 400–530 nm in wavelength is absorbed by the epidermis, where no vasculature exists. Instead, when using vascular filters, energy can be delivered more efficiently into the superficial and deep vessels [20]. Also, considering that the melanin extinction coefficient is high for 650–900 nm light, IPL-associated pain can be reduced. This will allow more focus on the vascular structures when using the vascular filter.

In dermatologic areas, IPL treatment targeting melanin is used therapeutically for hair removal via thermal damage to dermal papilla cells in hair follicles. The heat from the IPL treatment could possibly be absorbed by melanin, causing the loss of eyelashes [37]. Additionally, the melanin in the epidermis can cause hyper- or hypopigmentation following IPL treatment [38]. However, the use of a vascular filter can minimize energy absorbed by the epidermis and lower the possibility of hyper-or hypopigmentation by cutting the shorter wavelengths of light (400–530 nm) [29]. Pigmented irises can absorb light, which may subsequently cause serious ocular complications, from anterior uveitis to iris atrophy, secondary angle-closure glaucoma, and pupillary defects [39,40,41]. In one study, 14% of patients had adverse effects, including conjunctival cysts, floaters, hair loss, blistering, light sensitivity, and redness, leading 15% of them to terminate the IPL treatment [15]. Although protective measures, such as metal goggles and adhesive shields over the eyelids, help reduce adverse events, they may occasionally have to be removed or repositioned for adequate application of the IPL treatment to the target site. In our study, metal spatulas were used similarly, but there was no ocular or dermatologic adverse event observed in any patients [20].

Limitations in the present study include the absence of a control group. Hence, further studies with randomized controlled designs should be performed to confirm the current results. In addition, a comparison study evaluating the use of an acne versus a vascular filter, by applying one to each eye, is warranted to determine the effectiveness of vascular filters for the treatment of moderate and severe MGD. The present study did not perform non-invasive tear-film analysis although the tear-film lipid layer is a suitable parameter of MGD. Furthermore, the number of patients was rather small, and the follow-up period was short, as well. Therefore, further studies with larger sample sizes and longer follow-up periods are necessary to verify the long-term efficacy and safety of IPL treatment with a vascular filter. Lastly, only Fitzpatrick type 3 skin patients were enrolled. Because IPL’s efficacy and adverse events are influenced by skin type, further studies including various ethnic groups or skin types are required.

## 5. Conclusions

IPL treatment with a vascular filter could potentially be a replacement treatment modality in young patients and in those with a toothpaste-like meibum consistency in moderate to severe MGD, as evidenced by the findings that IPL with a vascular filter can safely and effectively relieve MGD signs and symptoms, especially the lid margin telangiectasia and ocular surface MMP-9 expression. Age and meibum consistency in the upper lid are important prognostic factors that can affect the clinical outcomes of patients undergoing IPL treatment with a vascular filter.

## Figures and Tables

**Table 1:** Clinical Signs and Symptoms before, and four weeks after, the last treatment of IPL, utilizing a vascular filter in patients with moderate to severe meibomian gland dysfunction.

Parameter	Before Treatment	4 Weeks after Last IPL Treatment	*p* Value ^1^
TBUT (s)	1.58 (0.86)	3.88 (1.35)	<0.001
SICCA staining score	5.98 (2.34)	1.98 (1.56)	<0.001
Oxford staining score	2.33(1.15)	1.03 (0.18)	<0.001
Schirmer’s test (mm)	7.06 (4.03)	7.58 (4.72)	0.438
Ocular irritation symptom score	2.98 (0.13)	1.17 (0.38)	<0.001

TBUT, tear film break-up time; SICCA, Sjögren’s International Clinical Collaborative Alliance; IPL, intense pulsed light. Results are presented as mean (standard deviation). ^1^ Statistical analysis was performed with a paired *t*-test.

**Table 2:** Lid margin telangiectasia, meibomian gland function, and MMP-9 positivity before and four weeks after the last IPL treatment using a vascular filter in patients with moderate and severe meibomian gland dysfunction.

Parameter		Before Treatment	4 Weeks after Last IPL Treatment	*p* Value ^1^
Lid margin telangiectasia	Upper eyelid (0–3)	2.71 (0.46)	1.36 (0.52)	<0.001
	Lower eyelid (0–3)	2.81 (0.40)	1.45 (0.54)	<0.001
Meibum color	Upper eyelid (0–3)	2.95 (0.22)	1.50 (0.50)	<0.001
	Lower eyelid (0–3)	2.97 (0.18)	1.50 (0.50)	<0.001
Meibum consistency	Upper eyelid (0–3)	2.60 (0.53)	1.33 (0.47)	<0.001
	Lower eyelid (0–3)	2.79 (0.45)	1.41 (0.56)	<0.001
MG grade		2.53 (0.54)	1.24 (0.56)	<0.001
MMP-9 positivity		90.2%	70.6%	0.013
MMP-9 level		1.92 (1.18)	1.24 (1.18)	<0.001

MG, meibomian gland; MMP-9, matrix metalloproteinase-9; IPL, intense pulsed light. Results are presented as mean (standarddeviation). ^1^ Statistical analysis was performed with paired *t*-test for continuous variables, and the McNemar test for categorical variables.

**Table 3:** Differences in baseline clinical parameters between the non-responder and responder groups.

Baseline Parameter		Non-Responder Group (*n* = 8)	Responder Group (*n* = 50)	*p* Value ^1^
Sex	Male: Female	3:5	13:37	0.672
Age (year)		68.5 (9.4)	62.4 (9.3)	0.008
TBUT (s)		1.88 (0.83)	1.54 (0.86)	0.310
SICCA staining score		4.88 (2.42)	6.16 (2.30)	0.015
Oxford staining score		2.00 (1.31)	2.38 (1.12)	0.388
MMP-9 level		1.00 (1.07)	2.09 (1.13)	0.015
Meibum color	Upper eyelid (0–3)	2.88 (0.35)	2.96 (0.20)	0.322
	Lower eyelid (0–3)	2.88 (0.35)	2.98 (0.14)	0.135
Meibum consistency	Upper eyelid (0–3)	2.00 (0.53)	2.68 (0.47)	0.008
	Lower eyelid (0–3)	2.25 (0.71)	2.86 (0.35)	0.045
Lid telangiectasia	Upper eyelid (0–3)	2.63 (0.52)	2.72 (0.45)	0.591
	Lower eyelid (0–3)	2.88 (0.35)	2.80 (0.40)	0.623

TBUT, tear film break-up time; SICCA, Sjögren’s International Clinical Collaborative Alliance; MMP-9, matrix metalloproteinase-9. Results are presented as mean (standard deviation). Non-responder group: no difference in meibomian gland grade; responder group: improvement in meibomian gland grade. ^1^ Statistical analysis was performed with independent *t*-tests for continuous variables, and Fisher’s exact test for categorical variables.

**Table 4:** Univariate analysis comparing odds ratios of baseline parameters to mean differences of meibomian gland grade in patients with moderate to severe meibomian gland dysfunction.

Baseline Parameter		Odds Ratio	95% Confidence Interval		*p* Value ^1^
			Lowest	Highest	
Sex	Female	1			
	Male	0.586	0.122	2.800	0.503
Age (year)		0.919	0.832	1.015	0.008
TBUT (s)		0.660	0.296	1.471	0.310
SICCA staining score		1.298	0.905	1.862	0.015
Oxford staining score		1.386	0.663	2.895	0.385
Meibum color	Upper eyelid (0–3)	3.429	0.274	42.956	0.339
	Lower eyelid (0–3)	7.000	0.392	125.040	0.186
Meibum consistency	Upper eyelid (0–3)	15.782	1.916	130.021	0.010
	Lower eyelid (0–3)	9.611	2.033	4.443	0.004
Lid margin telangiectasia	Upper eyelid (0–3)	1.543	0.325	7.333	0.586
	Lower eyelid (0–3)	0.571	0.063	5.193	0.619
MMP-9 level		2.856	1.130	7.219	0.027
Schirmer test		1.242	0.917	1.682	0.162

TBUT, tear film break-up time; SICCA, Sjögren’s International Clinical Collaborative Alliance; MMP-9, matrix metalloproteinase-9. ^1^ Statistical analysis was performed with univariate analysis using a logistic regression model.

**Table 5:** Multivariate analysis comparing odds ratios of baseline parameters to mean differences of meibomian gland grade in patients with moderate to severe meibomian gland dysfunction.

Baseline Parameter		Odds Ratio	95% Confidence Interval		*p* Value ^1^
			Lowest	Highest	
Age (year)		0.905	0.801	0.973	0.010
SICCA staining score		1.290	0.935	1.753	0.326
Meibum consistency	Upper eyelid (0–3)	10.826	0.404	290.417	0.010
	Lower eyelid (0–3)	3.747	0.192	73.089	0.384
MMP-9		1.855	0.578	5.951	0.299

SICCA, Sjögren’s International Clinical Collaborative Alliance; MMP-9, matrix metalloproteinase-9. ^1^ Statistical analysis was performed with multivariate analysis using a logistic regression model

## Data Availability

Not applicable.
